# Anti-dengue virus serotype 2 activity of tannins from porcupine dates

**DOI:** 10.1186/s13020-020-00329-7

**Published:** 2020-05-20

**Authors:** Lian Yih Pong, Peng Nian Yew, Wai Leng Lee, Yau Yan Lim, Syed Hassan Sharifah

**Affiliations:** 1 Jeffrey Cheah School of Medicine and Health Sciences, Monash University Malaysia, Jalan Lagoon Selatan, 47500 Bandar Sunway, Selangor Darul Ehsan Malaysia; 2 Infectious Diseases and Health Cluster, Tropical Medicine and Biology Platform, Monash University Malaysia, Jalan Lagoon Selatan, 47500 Bandar Sunway, Selangor Darul Ehsan Malaysia; 3 School of Science, Monash University Malaysia, Jalan Lagoon Selatan, 47500 Bandar Sunway, Selangor Darul Ehsan Malaysia; 4 Present Address: Department of Bioscience, Faculty of Applied Science, Tunku Abdul Rahman University College, Jalan Genting Kelang, 53300 Kuala Lumpur, Malaysia

**Keywords:** Porcupine dates, Antiviral agent, Dengue infection, Phytocompound, Traditional Chinese medicine, Natural product

## Abstract

**Background:**

Dengue fever is currently endemic in tropical and subtropical countries worldwide and effective drug against DENV infection is still unavailable. Porcupine dates, which are traditionally used to treat dengue fever, might contain potential anti-dengue compounds. Two porcupine dates, black date (BD) and powdery date (PD) from Himalayan porcupine (*Hystrix brachyura*), were investigated for their antiviral activities against DENV-2 in vitro.

**Methods:**

The methanol crude extracts (MBD and MPD) were prepared from the raw material of porcupine dates. The tannin-rich fractions (BDTF and PDTF) were isolated from their methanol crude extracts using column chromatography. The presence of tannins in BDTF and PDTF extracts was determined by fourier-transform infrared spectroscopy (FTIR) and nuclear magnetic resonance (NMR) analyses. The cytotoxicity and anti-DENV-2 activities including virus yield inhibition, virucidal, virus attachment and pre-treatment assays of the extracts were examined in Vero cells.

**Results:**

Our findings revealed that all the extracts of porcupine dates exhibited antiviral activity against DENV-2 in Vero cells. The IC_50_ of BDTF and PDTF were 25 µg/mL and 11 µg/mL respectively, while their methanol crude extracts demonstrated lower antiviral efficacy (IC_50_ ≈ 101–107 µg/mL). BDTF and PDTF also exerted a similar higher virucidal effect (IC_50_ of 11 µg/mL) than methanol crude extracts (IC_50_ ≈ 52–66 µg/mL). Furthermore, all the extracts inhibited the attachment of DENV-2 by at least 80%. Pre-treatments of cells with BDTF and PDTF markedly prevented DENV-2 infection when compared to methanol crude extracts.

**Conclusion:**

This study suggests that porcupine dates possess antiviral properties against DENV-2, which is attributed to its tannin compounds.

## Background

Dengue fever is the most prevalent mosquito-borne viral disease with high morbidity and mortality that is currently endemic in more than 100 countries within tropical and subtropical regions worldwide [1, 2]. It is caused by dengue virus (DENV) which is circulated as four distinct serotypes that are antigenic closely related, DENV-1, -2, -3 and -4. DENV is an enveloped, positive-sense single-stranded RNA virus that has been classified into genus of *Flavivirus* in the *Flaviviridae* family. The genome of DENV is approximately 11 kb in size and it encodes for three structural proteins, envelope (E), capsid (C) and premembrane (prM) proteins and seven non-structural proteins, NS1, NS2A, NS2B, NS3, NS4A, NS4B and NS5 [3]. DENV is transmitted to human by mosquito species of *Aedes aegypti* mainly as well as *Aedes albopictus* [4].

Nowadays, dengue is still a public health concern globally as it spreads rapidly worldwide and the incidence of dengue keeps increasing dramatically in recent decades. World Health Organization (WHO) has estimated 500,000 people would be diagnosed as severe dengue annually with a fatality rate of 2.5%. To date, there is no specific antiviral treatment for dengue illnesses. There is therefore, a compelling need to find and discover new drugs or compounds for dengue treatment. Traditional medicine has been used occasionally as a primary treatment for dengue fever, especially in Asian and African countries [5, 6]. Porcupine dates are one of the traditional medicines used by people in Southeast Asia to treat dengue fever by consuming orally the fine powder form of porcupine dates, and sometimes it has been used as multilateral treatment against various diseases including cancer and inflammation [7–9]. Porcupine dates are also known as porcupine bezoar, a phytobezoar that is formed from the partly digested plant materials or herbs in the stomach of Himalayan porcupine (*Hystrix brachyura*) [8, 10]. Many testimonies have claimed that porcupine dates can effectively cure dengue illness [9]; however, these claims lack scientific evidence and its relative mechanism of actions is still unknown.

In our previous study, two types of porcupine dates, the black date (BD) and powdery date (PD) were found to have a significant high content of total phenolics with 93% of tannins as major compounds [11]. In subsequent study, hydrolysable polygalloyl glucoses similar to commercial tannic acid ranging from 2 to 5 galloyl groups were identified in the tannins-fraction of BD and PD [12]. Tannin is an astringent large biomolecule that tends to bind and precipitate proteins, and it is known to be the main component in phytobezoar due to its agglomerating properties. In previous studies, tannin was found to possess antiviral activities against influenza A virus, human papillomavirus type 16, DENV, human cytomegalovirus (HCMV), hepatitis C virus (HCV), measles virus (MV), respiratory syncytial virus (RSV) and herpes simplex virus type 1 (HSV-1), in which these tannins effectively inhibited those infections by interfering the interaction between viral glycoprotein and cell surface glycosaminoglycans including heparan sulfate [13–15]. Apart from that, the oligomeric procyanidins derived from unripe apple peel, a condensed form of tannins, was found to induce innate antiviral immune responses against DENV infection in human peripheral blood mononuclear cell (PBMCs) [16].

In this study, we have hypothesized that the phytochemicals, particularly the hydrolysable tannins in porcupine dates have effective antiviral properties against DENV-2 by interfering the DENV-2 infectivity. The BD and PD from Himalayan porcupine (*Hystrix brachyura*) were investigated in this study in order to determine their antiviral activity against DENV-2. This study, for the first time, provides the scientific evidence on the anti-DENV property of the porcupine dates as well as its mode of action in inhibiting the DENV-2 in vitro. In addition, more in-depth nuclear magnetic resonance (NMR) analysis of the chemical content of porcupine dates was also reported for the first time.

## Methods

### Porcupine dates crude extraction

Raw material of porcupine dates, BD and PD obtained from a local Chinese medicinal shop were finely ground using pestle and mortar. One gram of samples were then extracted using 50 mL (1:50 w/v) of 100% methanol and shaken at 200 rpm using orbital shaker (Protech Model 719) for 1 h at room temperature. Extraction process was repeated three times using the same method, and the extracts were then pooled and subjected to rotary evaporation and lyophilized. The yields of the methanol crude extract were 48.2% for BD and 35.5% for PD. Two commercial tannic acids purchased from Sigma-Aldrich, ST and Friendemann Schmidt, FT were included as reference compounds in the screening of antiviral activity.

### Isolation of tannins from methanol crude extracts

Methanol crude extracts were subfractioned into ethanol soluble fraction using non-denatured absolute ethanol to remove the undesired compounds such as protein and non-phenolic phytochemicals. The ethanol fraction was subjected to column chromatography on Sephadex LH-20 (Sigma LH20100, 25–100 µm; 4 × 30 cm) using two mobile phases (solvent A: 95% EtOH: 5% H_2_O and solvent B: 70% acetone: 30% H_2_O). Samples were wet packed at the top of the column, and run through with three column volumes (≈ 350 mL) of solvent A. Eluent was checked with 2,2-diphenyl-1-picrylhydrazyl (DPPH) until no phenolic compound is eluted. Then, three column volumes (≈ 350 mL) of solvent B was used to elute the tannins bound to Sephadex LH-20 beads which were then collected as tannins fraction (sample of BDTF or PDTF). Eluted samples were subjected to rotary evaporation and lyophilized.

### Fourier-transform infrared spectroscopy (FTIR) analysis

FTIR analysis of BDTF, PDTF and ST tannic acid samples were done using FT-IR spectrometer (Perkin Elmer Spectrum Two). Approximately 5 mg of finely ground sample powder or standards was placed on the diamond ATR crystal of the spectrometer. Pressure was applied to the sample until the force gauge reached a value of 80. Triplicate of 15 scans were obtained for each sample.

### Nuclear magnetic resonance (NMR) analysis

^1^H-NMR spectra of BDTF, PDTF and ST tannic acid standard were obtained using Bruker High Resolution NMR Fourier 300 HD spectrometer with ^1^H NMR (300 MHz). Compounds were dissolved in DMSO-d solvent at 10 mg/mL for NMR analysis. Solvent peak by DMSO-d was identified at 2.5 ppm.

### Cell line and dengue virus

Vero cells (African green monkey kidney) were maintained in minimum essential media (MEM) supplemented with 10% FBS, 1% HEPES and 1% penicillin–streptomycin antibiotic (Gibco) at 37 °C in a humidified incubator with 5% CO_2_. Dengue virus serotype 2 (DENV-2), which was isolated from dengue-infected patient serum, was used in this study. The serotype of this clinical isolate was confirmed by whole genome sequencing (GenBank accession no. MH488959). Dengue virus stock was prepared by propagation in Vero cells. The virus titer was determined via focus formation assay in Vero cells.

### Cytotoxicity assay

Vero cells grown in 96-well plates were incubated with 100 µL of growth medium containing various concentrations of extracts or tannic acids for 48 h at 37 °C in a humidified incubator with 5% CO_2_. Thereafter, 10 µL of MTT reagent was added at final concentration of 0.5 mg/mL and cells were further incubated for 4 h. An equal amount of solubilization solution (10% SDS in 0.01 N HCl) was added and plate was further incubated overnight. The optical density was read at 570 nm with a background absorbance subtracted at 690 nm. The cell viability rate was plotted against various concentrations of compounds to determine the cytotoxic concentration 50% (CC_50_) that causes 50% reduction in cell viability.

### Virus yield inhibition assay

Vero cells grown in 96-well plates were infected with DENV-2 at multiplicity of infection, MOI of 0.5 for 1 h at 37 °C. Thereafter, the inoculums were removed and cells were rinsed once with PBS. Infected cells were then incubated with various concentrations of extracts or tannic acids for 48 h at 37 °C in a humidified incubator with 5% CO_2_. After 48 h of post-infection, supernatants of infected cells were harvested and subjected to focus formation assay in duplicate. The inhibitory concentration 50% (IC_50_) was determined by plotting the percentage of virus yield reduction against various concentrations of extracts.

### Focus formation assay (FFA)

FFA was performed as previously described [17] and according to the WHO guidelines [18] with some modifications. The supernatants containing virus particles were serially diluted in tenfold. Vero cells (2 × 10^5^ cells) grown in 24-well plates were inoculated with 100 µL of inoculum followed by infection for 1 h at 37 °C. After 1 h of virus adsorption, inoculums were removed completely and 2% CMC (carboxymethyl cellulose) overlay medium containing MEM, 2% FBS, 1% penicillin–streptomycin antibiotic and 1% HEPES was overlaid. The plates were incubated at 37 °C in a humidified incubator with 5% CO_2_ for 4 days.

On Day 4 of post-infection, immunostaining against dengue virus was performed. The overlay medium was removed and cell monolayers were washed twice with wash buffer (Tris-buffered saline containing 0.1% Tween-20, TBST). After fixation with cold 80% acetone followed by washing, the cells were incubated with blocking buffer (wash buffer containing 1% BSA and 0.5% Triton X-100) at 37 °C for 45 min. Cells were then incubated with mouse monoclonal dengue virus type 1, 2, 3 and 4 antibody [D1-11(3)] (Genetex) at 37 °C for 1 h. After washing, alkaline phosphatase-conjugated rabbit anti-mouse IgG (Santa Cruz) or alkaline phosphatase-conjugated goat anti-mouse IgG (Genetex) was added for 1 h at 37 °C followed by washing. The foci were visualized by adding a mixture of NBT (nitrotetrazolium blue chloride) and BCIP (5-bromo-4-chloro-3′-indolyphosphate p-toluidine salt) substrates (Bio Basic). The number of foci was counted and percentage of focus reduction was calculated.

### Virucidal assay

An equal volume of various concentrations of extracts or tannic acids serially diluted in fourfold starting at non-toxic concentration were incubated with 30 foci-forming units (FFU) of DENV-2 for 1 h at 37 °C. Thereafter, 100 µL of extract-virus mixture was inoculated on Vero cells grown in 24-well plates for 1 h at 37 °C. After incubation, the inoculums were replaced by 2% CMC overlay medium. The plates were incubated at 37 °C in a humidified incubator with 5% CO_2_ for 4 days. The immunostaining against dengue virus was performed as described in FFA. The IC_50_ values were determined as mentioned above.

### Virus attachment assay

The virus attachment assay was performed as previously described with some modifications [19]. Vero cells grown in 24-well plates were infected with 30–50 FFU of DENV-2 in the presence or absence of BDTF, PDTF, methanol crude extracts, tannic acids of FT and ST at maximum non-toxic concentration (MNTC) of 100 µg/mL, 50 µg/mL, 200 µg/mL, 50 µg/mL and 25 µg/mL respectively for 1 h at 4 °C. Heparin (Sigma-Aldrich) was tested at 800 µg/mL as a control in this assay. After 1 h of virus infection, the inoculum was removed and cells were washed twice with cold PBS. Cells were then covered with 2% CMC overlay medium and incubated at 37 °C in a humidified incubator with 5% CO_2_ for 4 days. The immunostaining against dengue virus was performed as described in FFA.

### Pre-treatment assay

Vero cells grown in 24-well plates were incubated with the extracts or tannic acids at MNTC for 1 h at 37 °C. A control, heparin (Sigma-Aldrich) at 800 µg/mL was included. Thereafter, the compounds were removed completely followed by infection with 40 FFU of DENV-2 for 1 h at 37 °C. After 1 h of virus infection, the inoculum was replaced by 2% CMC overlay medium. The plates were incubated at 37 °C in a humidified incubator with 5% CO_2_ for 4 days. The immunostaining against dengue virus was performed as described in FFA.

### Statistical analysis

The data were subjected to statistical analysis using ordinary one-way ANOVA followed by Dunnett’s multiple comparison test, unless otherwise stated. The nonlinear regression with a model of sigmoidal dose–response was used to determine all the CC_50_ and IC_50_ values except the IC_50_ value of MBD. The IC_50_ value of MBD was determined from the graph that was plotted by the percentage of virus yield reduction against the extract’s concentrations of 50 and 200 μg/mL by using linear regression, as there was a lack of dose–response effect for MBD. The analyses were performed with GraphPad Prism 7 software (version 7.02). The results are expressed as the mean ± SEM of two independent experiments. The p values less than 0.05 were defined as statistically significant.

## Results

### Determination of the tannin compounds in BDTF and PDTF extracts

**Fig. 1:**
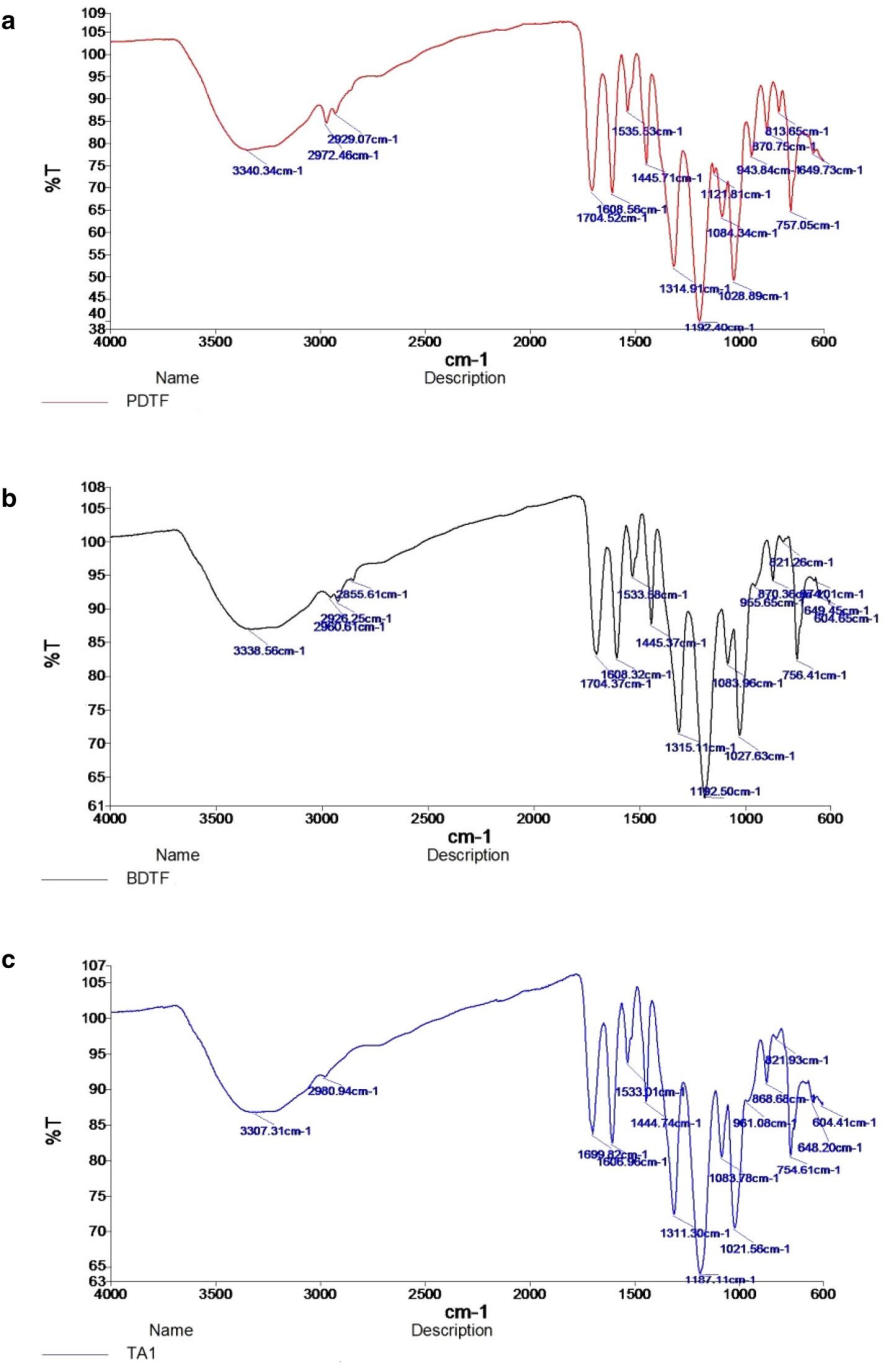
FTIR spectra of **a** BDTF, **b** PDTF and **c** ST tannic acid. Finely ground sample powder was placed on the diamond ATR crystal of the FTIR spectrometer. Pressure was applied to the sample with 80 N force. The spectra represent the average of 15 scans

**Fig. 2:**
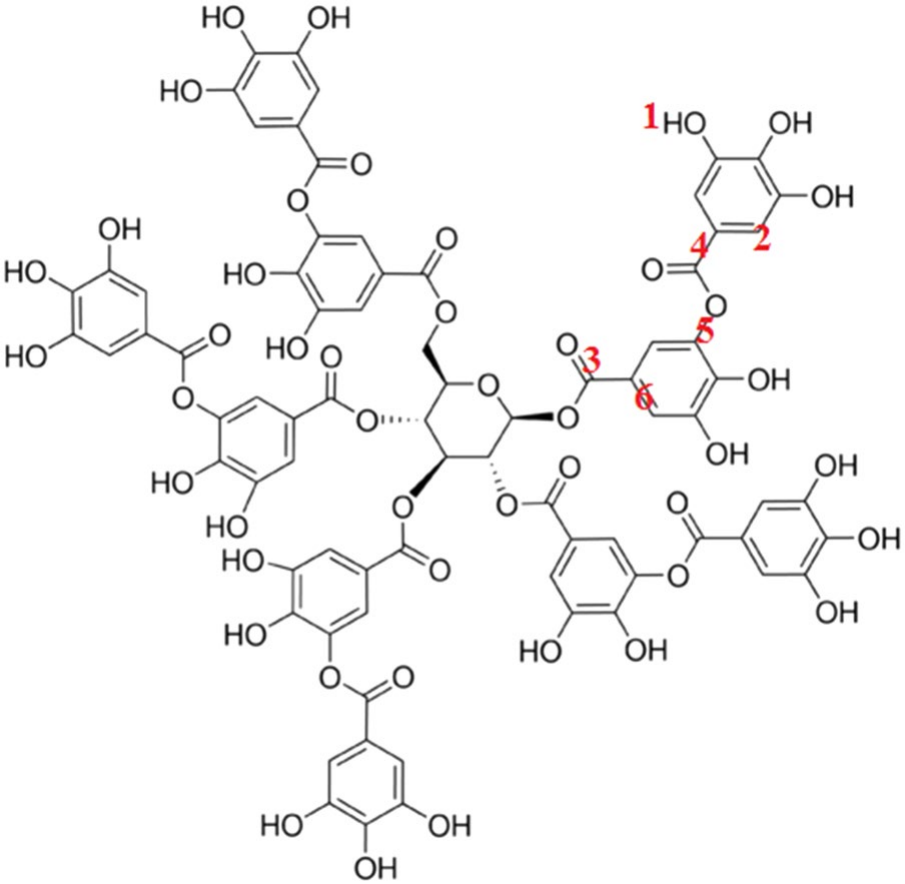
Illustration of the distinct functional group in tannic acid detected by FTIR analysis. Labelled numbers correspond to various wavenumber of functional group detected using FTIR analysis as summarized in Table 1. Tannic acid and its derivatives are putatively identified in BDTF and PDTF

FTIR was used to identify the organic functional groups present in fractionated samples. The FTIR spectra of both BDTF and PDTF highly matched to that of commercial tannic acid, which all have major bands at wavenumbers around 3340, 2926–2972, 1700–1718, 1608, 1533, 1445, 1315, 1192, 1083, 1027, 870 and 756 cm^−1^ (Fig. 1). In view of the tannins as reported in literature [20], the functional group attributed to different positions of the galloyl group of tannic acid are depicted and summarized (Fig. 2 and Table 1). Based on the FTIR results, it is suggested that the tannin compounds present in BDTF and PDTF are essentially tannic acid or its derivatives. However, some minor differences in the spectra such as 1121 and 813 cm^−1^ bands in BDTF were not observed in commercial tannic acid standard. This suggests that BDTF and PDTF are slightly structurally different from the commercial tannic acid, possibly with minor substitution of the side group.

**Table 1:** FTIR wavenumber and allocated functional groups of reported tannins, BDTF, PDTF and tannic acid standard (ST)

No.	Functional group	Wavenumber (cm^−1^)			
		Tannins^a^	BDTF	PDTF	Tannic acid (ST)
1	OH	3356	3339	3340	3307
2	C–H	2725	2926; 2972	2929; 2972	2980
3	C=O (ester)	1718	1704	1705	1700
4	C–C_arom_	1614; 1452	1608; 1445	1608; 1446	1607; 1445
5	C–O	1191	1193	1192	1187
6	C_arom_–H (out of plane bending)	754	756	757	754

^a^The data of reported tannins are obtained from a previous study done by Pantoja-Castro and González-Rodríguez [20]

**Fig. 3:**
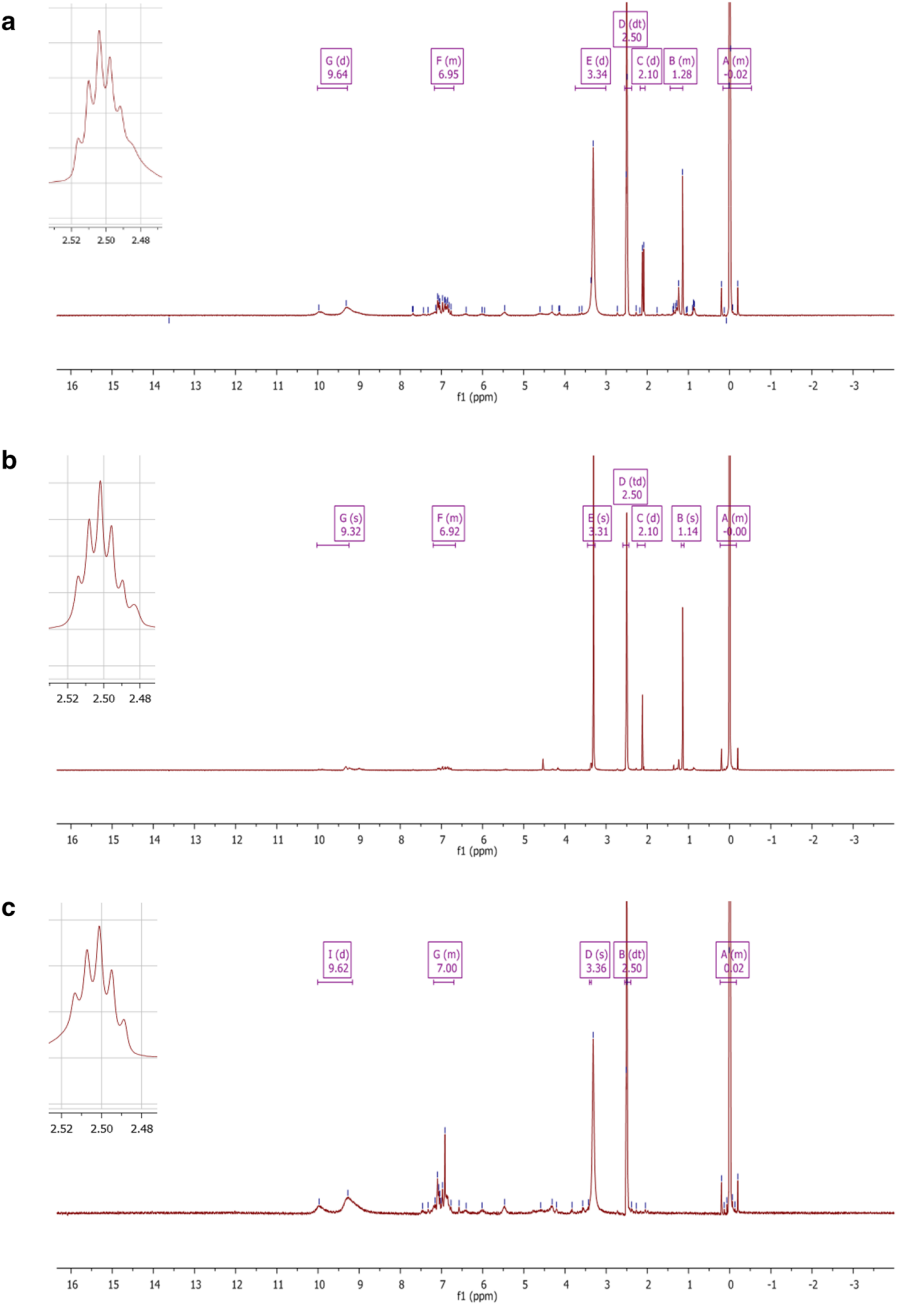
^1^H-NMR spectra of **a** BDTF, **b** PDTF and **c** ST tannic acid. Lyophilized samples were dissolved in deuterated DMSO at 10 mg/mL. The proton shift of each sample was obtained using Bruker High resolution NMR Fourier 300 HD spectrometer (300 MHz). Similarity of major chemical shifts was observed in all three spectra

Based on the comparison of proton NMR spectra, the major chemical shifts in BDTF and PDTF are similar to that of tannic acid standard (Fig. 3 and Table 2). The similarity of major proton chemical shifts further suggests the presence of tannic acids in BDTF and PDTF fractions. In a recent study, both BDTF and PDTF have been structurally determined using high-performance liquid chromatography (HPLC) and ESI tandem mass spectroscopy (ESI–MS/MS) [12]. The fragmentation pattern of respective mass spectrum suggests the presence of several hydrolyzed forms of tannins with varying number of galloyl groups. Collectively, this is the first time compound analysis of porcupine dates has ever been reported.

**Table 2:** Chemical shift of BDTF, PDTF and ST tannic acid in ^1^H-NMR analysis

Peak	Chemical shift (δH/ppm)		
	BDTF	PDTF	ST tannic acid
1	1.28 (m)	1.14 (s)	
2	2.10 (d)	2.10 (d)	
3	2.50 (dt)	2.50 (td)	2.50 (dt)
4	3.34 (d)	3.31 (s)	3.36 (s)
5	6.95 (m)	6.92 (m)	7.00 (m)
6	9.64 (d)	9.32 (s)	9.62 (d)

### Inhibitory effects of porcupine dates extracts on DENV-2 infection

**Fig. 4:**
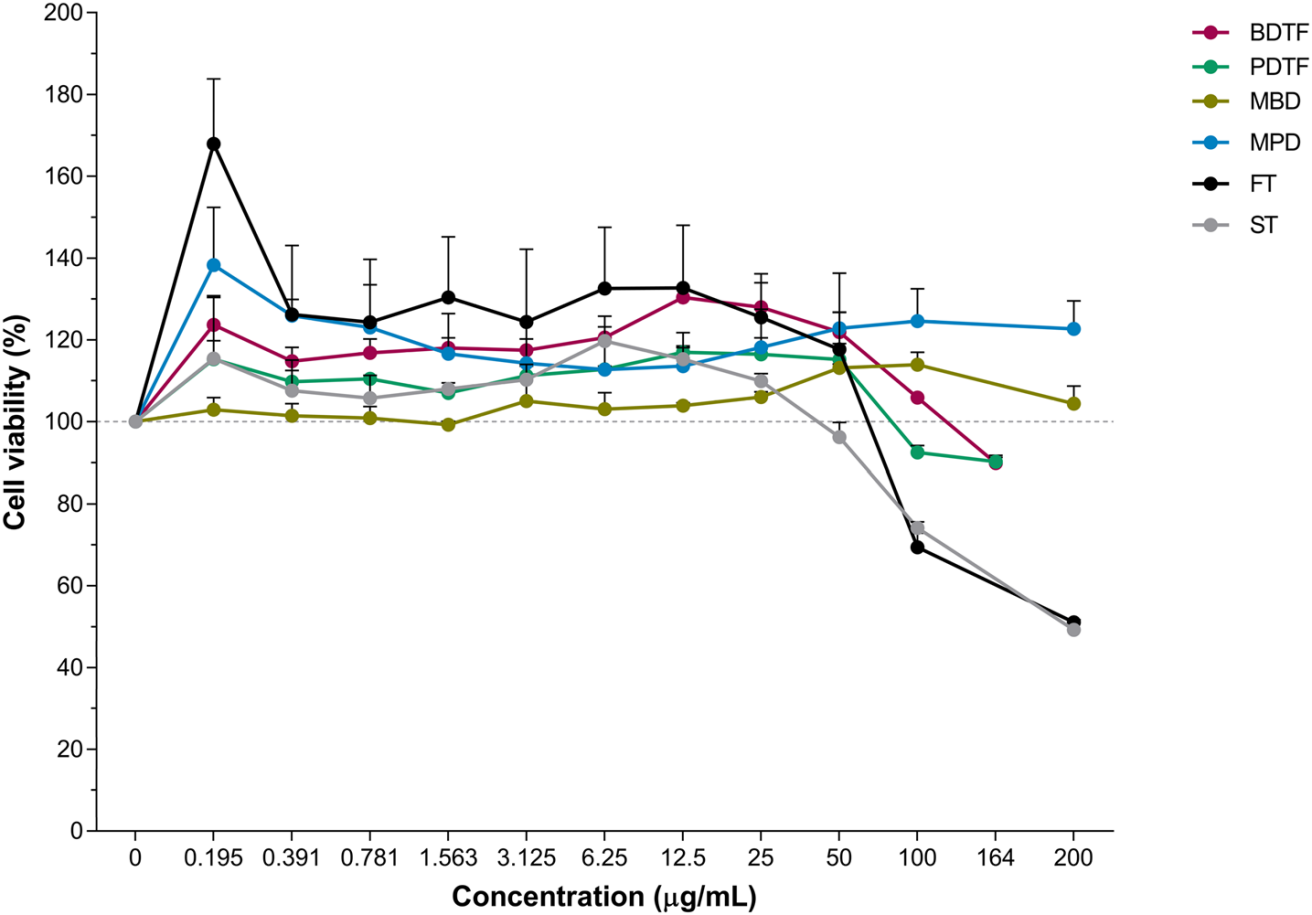
Cytotoxicity effect of porcupine dates extracts and tannic acids on Vero cells. The methanol crude extracts (MBD and MPD) and tannic acid reference compounds (FT and ST) were evaluated at concentration of 200 µg/mL down to 0.195 µg/mL, while BDTF and PDTF were evaluated at concentration of 164 µg/mL down to 0.195 µg/mL. Vero cells were incubated with various concentrations of extracts or tannic acids for 48 h at 37 °C. After that, cell viability was assessed by MTT assay. Values represent the mean of duplicate assays ± SEM

**Fig. 5:**
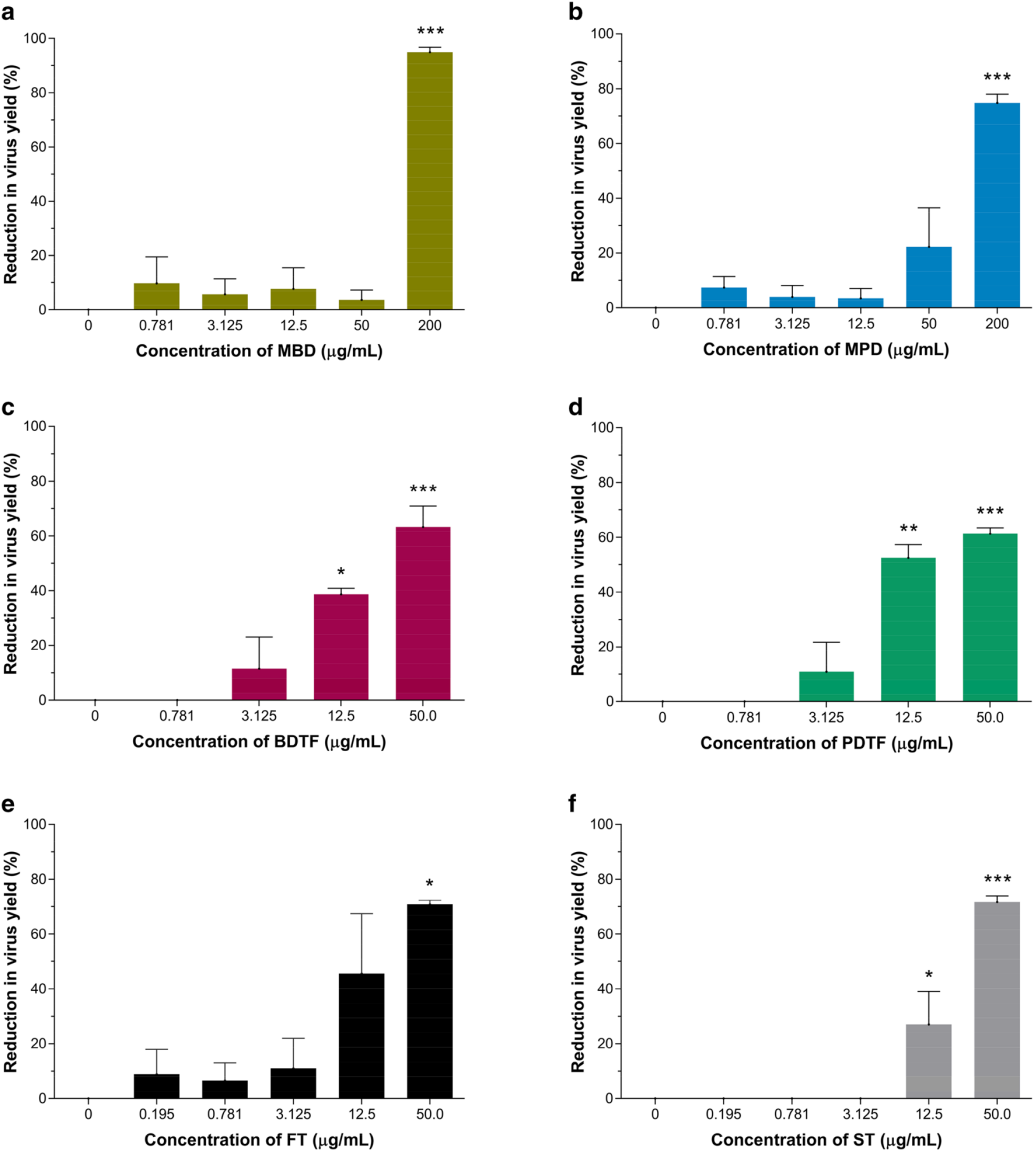
Inhibitory effects of porcupine dates extracts and tannic acids on DENV-2 in Vero cells. Vero cells were infected with DENV-2 at MOI of 0.5 for 1 h at 37 °C and the cells were treated with indicated concentrations of **a** MBD, **b** MPD, **c** BDTF, **d** PDTF extracts or **e**, **f** tannic acids. After 48 h of post-infection, the supernatants of infected cells were harvested and subjected to focus formation assay in duplicate to determine the virus yield. The data were subjected to statistical analysis using ordinary one-way ANOVA followed by Dunnett’s multiple comparison test. Values represent the mean ± SEM of two independent experiments performed in duplicate. *p < 0.05, **p < 0.01, ***p < 0.001

**Fig. 6:**
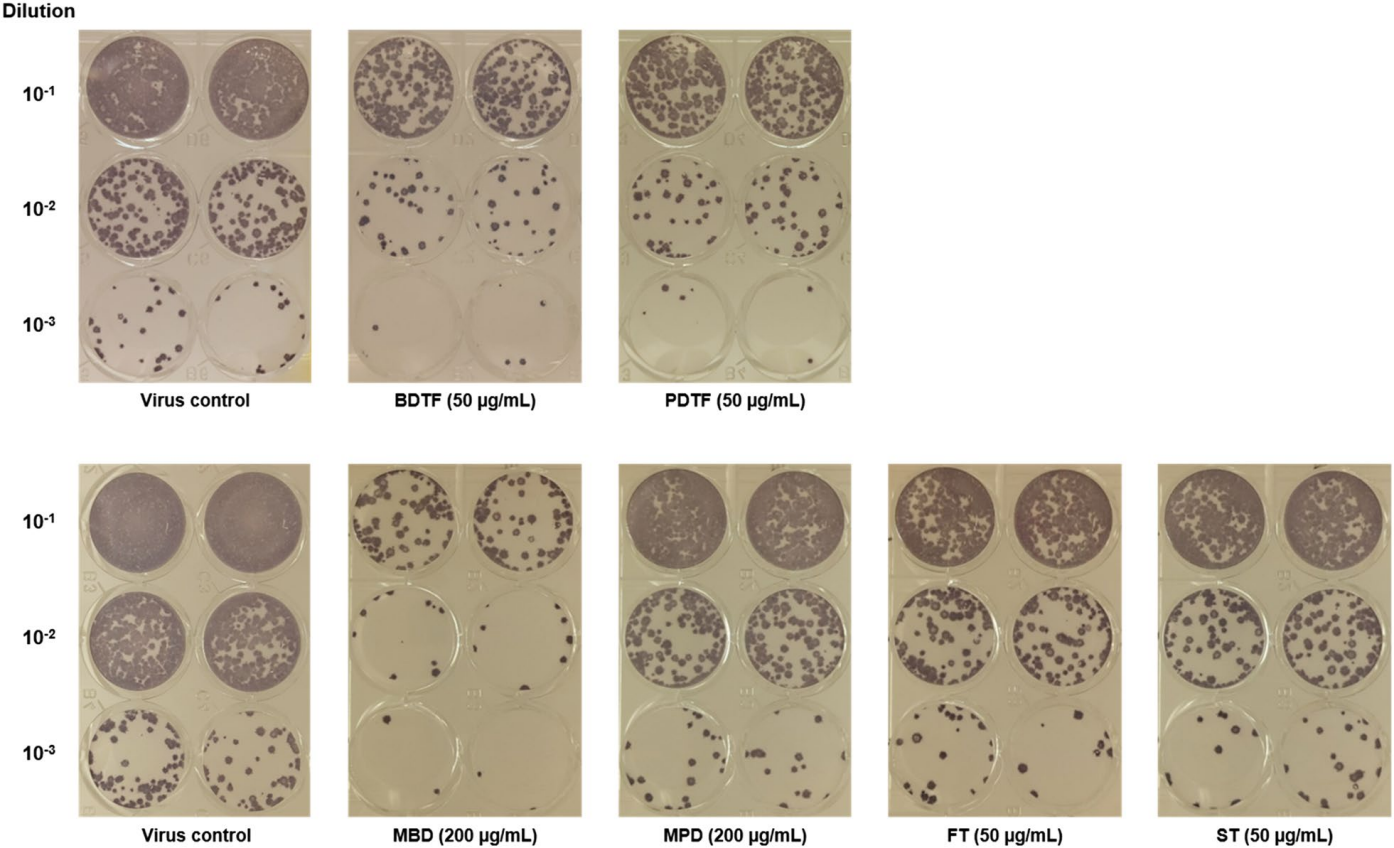
Representative focus formation of DENV-2 in Vero cells. DENV-2 infected-Vero cells were treated with porcupine dates extracts or tannic acids at non-toxic concentration and the virus yield was determined via focus formation assay in duplicate

Cytotoxicity of methanol crude extracts (MBD and MPD) and tannin fractions of black date (BDTF) and powdery date (PDTF) were first analysed in Vero cells to establish appropriate concentrations for further studies. To evaluate the inhibitory effect of porcupine dates on DENV-2, the DENV-2-infected Vero cells were treated with various types of porcupine dates extracts at various non-toxic concentrations. As illustrated in Fig. 4, both methanol crude extracts, BDTF and PDTF were not toxic to Vero cells at all concentrations up to 200 µg/mL, 100 µg/mL and 50 µg/mL, respectively. MBD and MPD exhibited similar lower antiviral activity against DENV-2 with an IC_50_ of 101 and 107 µg/mL respectively, and a selectivity index (SI) of more than 2 when compared to both BDTF and PDTF (Fig. 5 and Table 3). However, the inhibition of virus yield by MBD was not in a dose-dependent manner, thus the IC_50_ of MBD may not be accurate. In contrast, BDTF exhibited its antiviral activity against DENV-2 in a dose-dependent manner with an IC_50_ of 25 µg/mL and an SI of more than 7 (Fig. 5c and Table 3). PDTF also showed similar inhibition toward DENV-2 but with a greater antiviral activity; as little as of 11 µg/mL of PDTF caused a 50% inhibition in DENV-2 yield, resulting in a higher SI of more than 15 when compared to BDTF (Fig. 5d and Table 3). These results suggest that PDTF exhibited greater antiviral activity against DENV-2 than BDTF. To determine whether the antiviral activity observed in the porcupine dates is attributed to the tannins in those porcupine dates, two commercial tannic acids, FT and ST were included as reference compounds. Both FT and ST were non-cytotoxic at all concentrations up to 25 µg/mL (Fig. 4). FT inhibited the DENV-2 replication with an IC_50_ of 18 and an SI of more than 11, whilst ST exhibited its antiviral activity with an IC_50_ of 26 µg/mL and an SI of 8 (Fig. 5 and Table 3). The formation of DENV-2 focus on Vero cells treated with porcupine dates extracts or tannic acids is shown in Fig. 6.

**Table 3:** IC_50_, CC_50_ and selectivity indices of porcupine dates extracts and tannic acids against DENV-2 in Vero cells

Sample	CC_50_ (µg/mL)	IC_50_ (µg/mL)^b^	Selectivity index (SI)^c^
MBD	NC^a^	101 ± 10^d^	> 2
MPD	NC^a^	107 ± 24	> 2
BDTF	> 164	25 ± 7	> 7
PDTF	> 164	11 ± 3	> 15
FT	> 200	18 ± 15	> 11
ST	196	26 ± 8	8

^a^NC: No cytotoxicity at all concentration up to 200 µg/mL tested against Vero cells

^b^Each value represents the mean ± SD of two independent experiments performed in duplicate

^c^Selectivity index was calculated by dividing CC_50_ by IC_50_

^d^IC_50_ of MBD may not be accurate as it was not in a dose-dependent manner. It was estimated from the plot of the percentage of virus yield reduction against the extract’s concentrations of 50 and 200 μg/mL, by using linear regression

### Virucidal activity of porcupine dates extracts against DENV-2

**Fig. 7:**
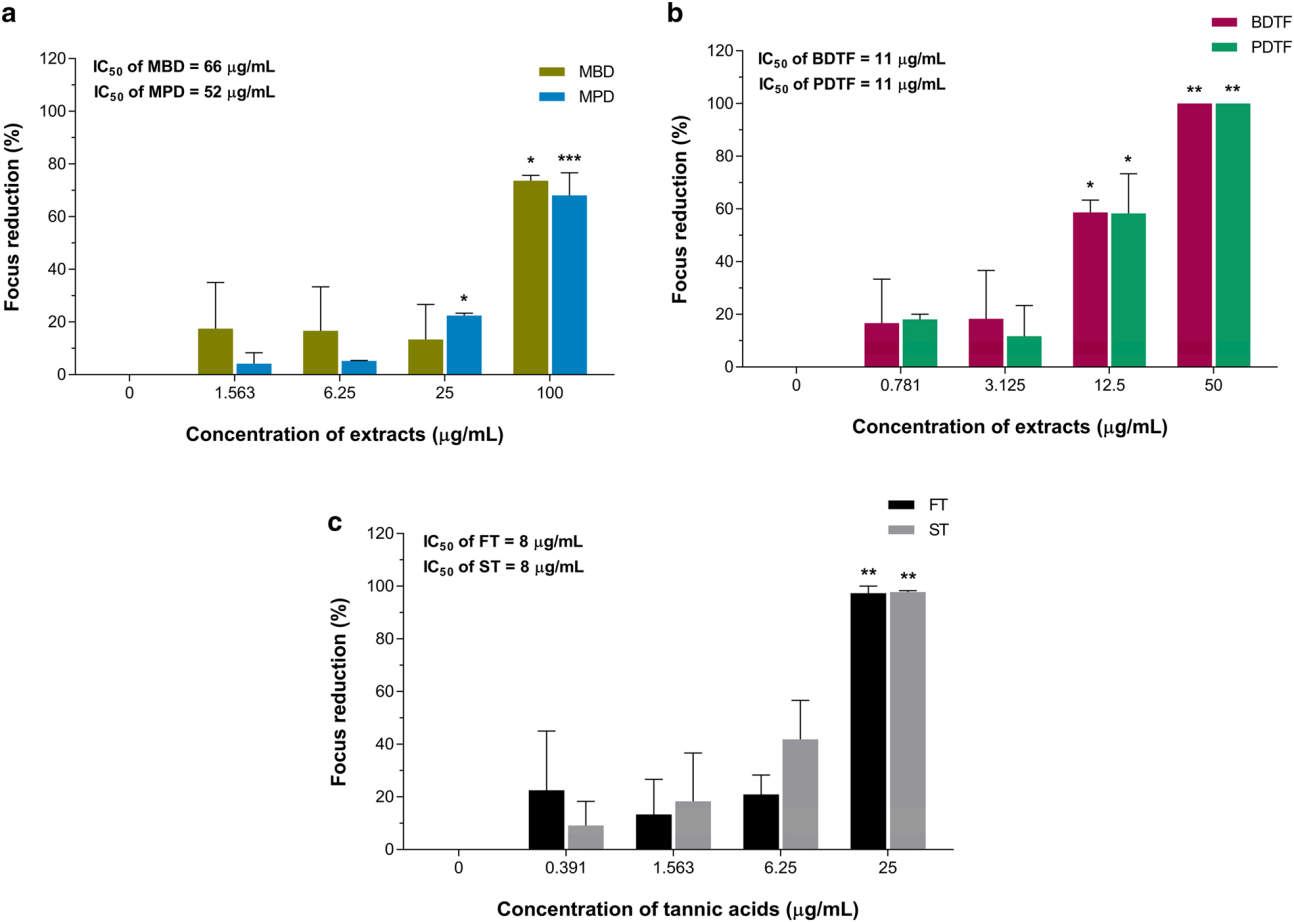
Virucidal effects of porcupine dates extracts and tannic acids on DENV-2 in Vero cells. The indicated concentrations of **a** methanol crude extracts, **b** tannin fractions of BD and PD, or **c** tannic acids were incubated with 30 FFU of DENV-2 for 1 h at 37 °C. The compound-virus mixture was then incubated with Vero cells and the mixture was removed after 1 h of infection. Cells were covered with overlay medium and incubated for 4 days. The number of foci was determined and counted via focus-formation assay. Data were subjected to statistical analysis using ordinary one-way ANOVA followed by Dunnett’s multiple comparison test. Values represent the mean ± SEM of two independent experiments performed in duplicate. *p < 0.05, **p < 0.01, ***p < 0.001

To evaluate the virucidal effect of porcupine dates on DENV-2, the porcupine dates extracts at various non-toxic concentrations were incubated with the DENV-2 directly and the ability of virus to replicate and infect was assessed via viral focus formation assay. MBD and MPD exhibited their dose-dependent virucidal activity against DENV-2 with an IC_50_ of 66 and 52 µg/mL respectively, and these extracts inactivated the DENV-2 significantly by 73.7% and 68.1% at concentration of 100 µg/mL, respectively (Fig. 7a). In contrast to MBD and MPD, BDTF and PDTF demonstrated an identical higher virucidal effect toward DENV-2 with an IC_50_ of 11 µg/mL (Fig. 7b). Based on the IC_50_ values, the virucidal effects of BDTF and PDTF were approximately sixfold and fivefold higher than the MBD and MPD, respectively. In addition, both BDTF and PDTF exterminated the DENV-2 replication at concentration of 50 µg/mL significantly and a 58% of significant virus inhibition was observed in both extracts at 12.5 µg/mL. Intriguingly, both FT and ST also exerted comparable virucidal effect on DENV-2 replication in a dose-dependent manner with a similar IC_50_ of 8 µg/mL, in which the virus yield was decreased significantly by at least 97% at concentration of 25 µg/mL (Fig. 7c).

### Effect of porcupine dates extracts on DENV-2 attachment on host cells

**Fig. 8:**
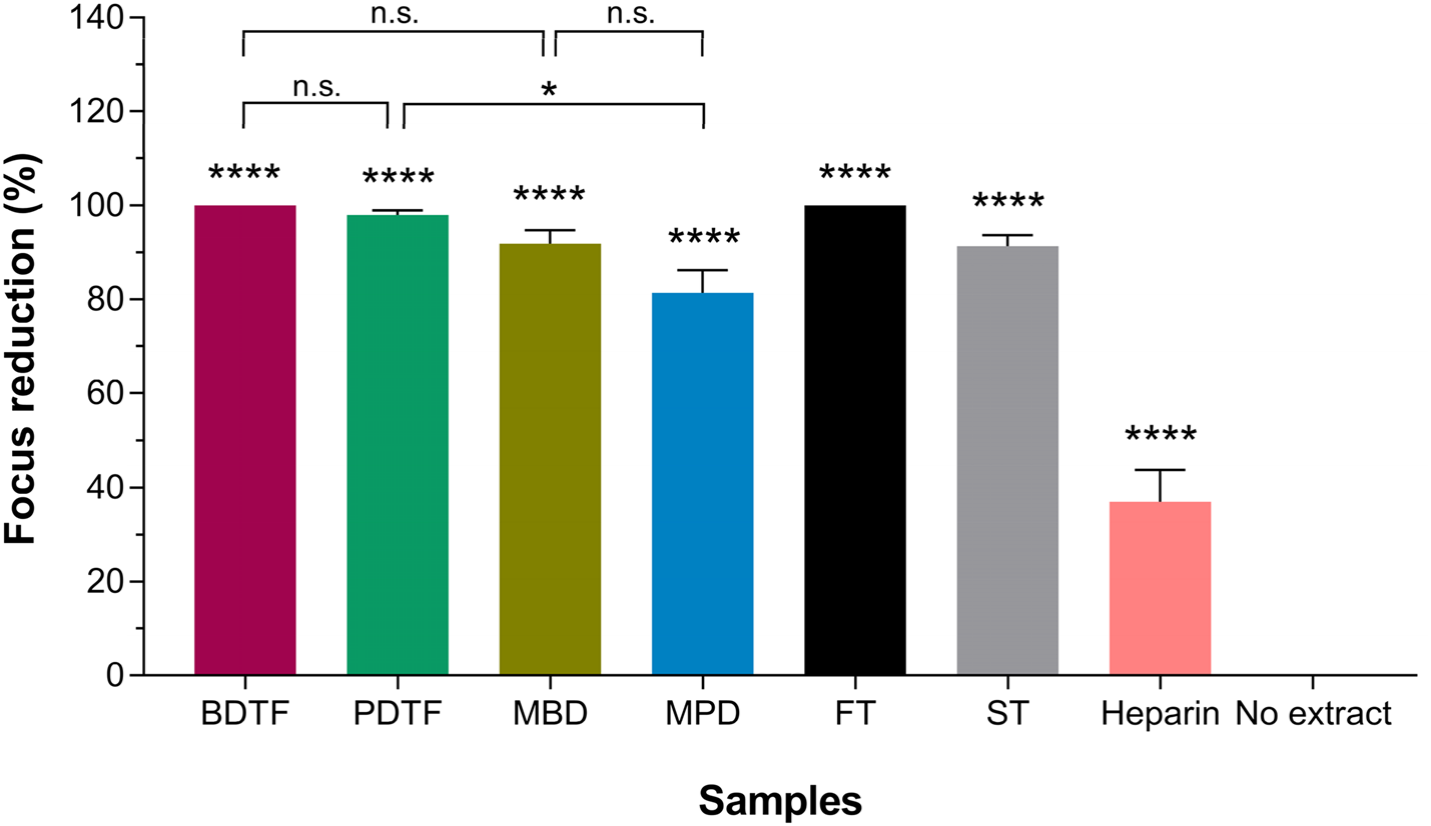
Effect of porcupine dates extracts and tannic acids on DENV-2 attachment in Vero cells. The extracts or tannic acids were incubated with Vero cells at MNTC together with 30–50 FFU of DENV-2 for 1 h at 4 °C. Heparin was assayed at 800 µg/mL as a control. The mixture containing unadsorbed virus was replaced with overlay medium after washing. After 4 days of incubation, the cells were subjected to immunostaining against DENV. Data were statistically analysed using ordinary one-way ANOVA followed by Dunnett’s and Sidak’s multiple comparison tests. Values represent the mean ± SEM of two independent experiments performed in duplicate. *p < 0.05, ****p = 0.0001, “n.s.” indicates not significant

To determine whether the porcupine dates extracts able to block the early step of DENV replication, which is the virus attachment, the extracts were evaluated at MNTC by incubating together with DENV-2 on Vero cells at 4 °C. All the porcupine dates extracts significantly reduced the formation of viral foci by at least 80% (Fig. 8). Remarkably, BDTF was found to 100% inhibit the DENV-2 infection at 100 µg/mL, while PDTF-treated DENV-2 infection was reduced by 98.4% at 50 µg/mL. Similar results were observed in FT and ST tannic acids with an inhibition of 100% and 93.1%, respectively. Furthermore, the antiviral activity of tannin fractions of BD and PD were found significantly higher than the methanol crude extracts, MBD and MPD by 6.3% and 13.3%, respectively. These results demonstrated that these porcupine dates extracts are active inhibitors against the adsorption step of DENV-2 and this antiviral activity most probably attributed to the tannin compounds in porcupine dates. Heparin, a known active inhibitor against virus entry of DENV [13, 21–23], was also examined as a control in this assay. Heparin was found to be less potent in inhibiting the attachment of DENV-2 to Vero cells, in which 37% of virus inhibition was observed at the highest concentration been tested in this study (800 µg/mL). Nonetheless, this result shows that glycosaminoglycans on cell surface are important for the DENV-2 attachment to host cells [21, 22].

### Pre-treatment effect of porcupine dates extracts on DENV-2 infection

**Fig. 9:**
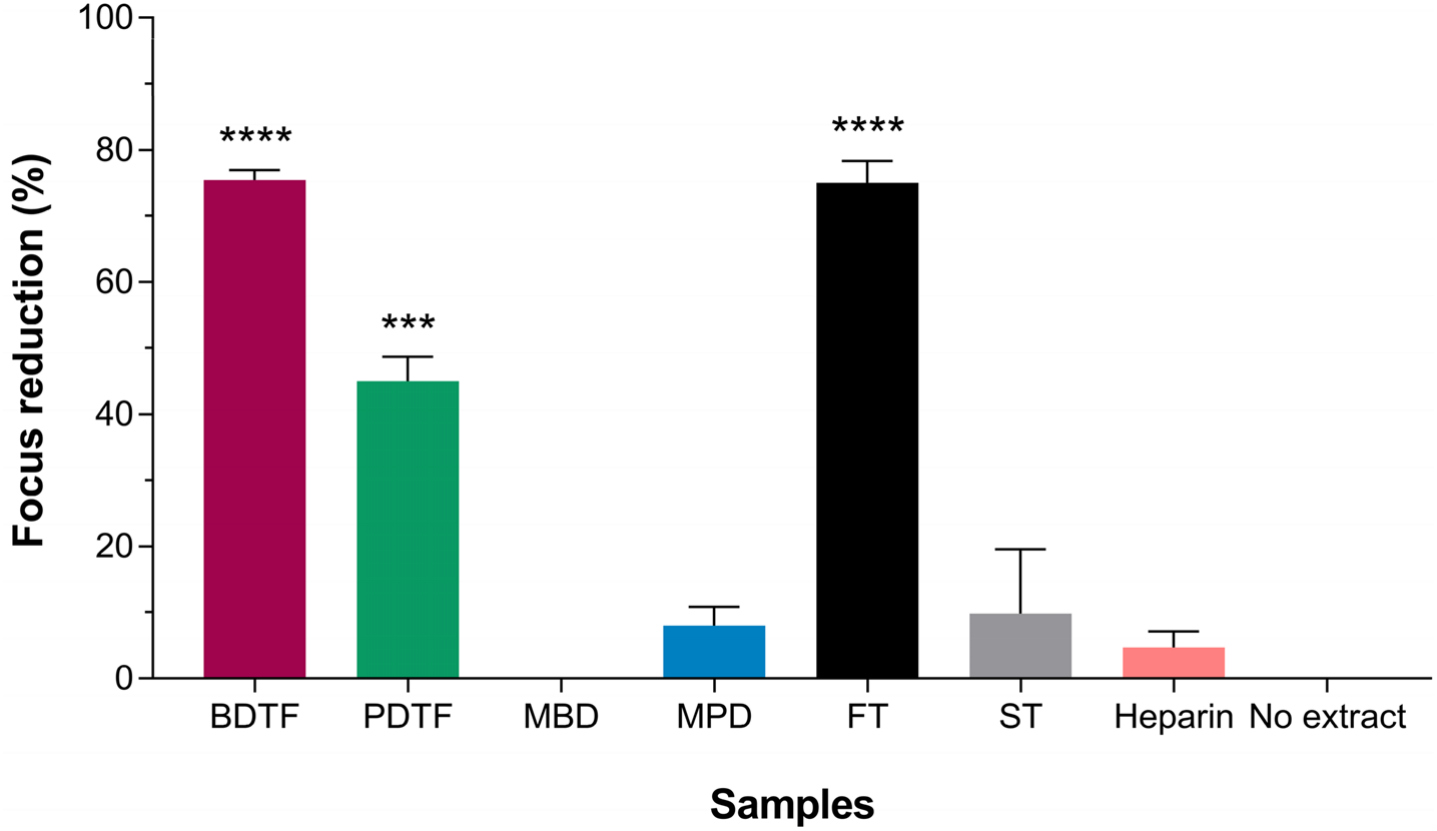
Effect of pre-treatment with porcupine dates extracts and tannic acids on DENV-2 infection in Vero cells. The individual extracts or tannic acids were incubated with Vero cells at MNTC for 1 h at 37 °C prior to infection with 40 FFU of DENV-2 for 1 h. Heparin was assayed at 800 µg/mL as a control. The inoculum was then replaced with overlay medium. The cells were subjected to immunostaining against DENV after 4 days of incubation. Data were statistically analysed using ordinary one-way ANOVA followed by Dunnett’s multiple comparison test. Values represent the mean ± SEM of two independent experiments. ***p < 0.001, ****p = 0.0001

The pre-treatment antiviral effect of porcupine dates extracts was examined by incubating the Vero cells with the extracts at MNTC for 1 h at 37 °C prior to DENV-2 infection, in order to determine if there is any direct interaction between the extracts and host cells that contribute to the antiviral action. Pre-treating the cells with BDTF was found to inhibit the virus infection by 75.4% significantly and similar result was observed in FT tannic acid, which caused inhibition of 75.1% significantly (Fig. 9). However, pre-treatment with PDTF exhibited lower antiviral activity when compared to BDTF, which resulted in a significant virus inhibition of 45.0%. In contrast, the antiviral activity of the pre-treatment with MPD or ST showed only 8.0–9.8% of inhibition and there was no virus inhibition observed when pre-treats the cells with MBD (Fig. 9). Pre-treating the cells with heparin at 800 µg/mL had no significant inhibitory effect on the infectivity of DENV-2, which is similar to the previous result attained by Hung et al. [22] in BHK cells. These results suggest that the tannins in BDTF and PDTF could be the key compounds that promote the antiviral effect. The pre-treatment of cells with BDTF evidently demonstrated the highest antiviral activity among the other extracts, indicating that this extract could efficiently block the virus attachment to the host cells.

## Discussion

Apart from the development of dengue vaccine, one of the alternative ways to fight against dengue infection is through the discovery of active compounds or natural products that are specifically directed at killing the virus or induce host innate immune responses against dengue infection without enhancing humoral responses which might cause complications in patient. Currently, there is no active compound or alternative medicine available for treating dengue illnesses. Therefore, it is of utmost importance that research in discovering anti-DENV treatments for human is expedited. Some natural products like marine seaweeds and approved drugs have been shown to inhibit DENV-2 replication in vitro [19, 24–28]; however, their effectiveness in combating dengue infections in human or in vivo has not been determined. Unlike these compounds, another natural product, the porcupine dates have long been used as a traditional Chinese medicine to treat dengue illnesses in human in some of the Southeast Asian countries [9].

In this study, methanol crude extract of porcupine dates (MBD and MPD) were shown as active DENV-2 inhibitors that can inhibit the infectivity of internalized DENV-2 in Vero cells and extracellular DENV-2 particles, providing evidence that porcupine dates could be used to treat DENV-2 infection. Both MBD and MPD were non-toxic to Vero cells up to 200 μg/mL, which was similar to a previous study done on normal human colon cells [12]. DENV-2 was selected as the main target for the antiviral activity of porcupine dates in this study because most cases of secondary infection by DENV-2 manifested a more severe form of dengue due to the antibody-dependent enhancement (ADE) of infection exerted by the host immune system responses, when compared to the other serotypes [29–32]. Moreover, DENV-2 has been shown to be the predominant strain among the other serotypes (DENV-1, DENV-3 and DENV-4) in causing dengue in many endemic countries [33–35]. Furthermore, to date, the search for an effective and efficacious vaccine is continuing. The efficacy of the first licensed dengue vaccine i.e. Dengvaxia (CYD-TDV) against DENV-2 is still at its lowest with an efficacy of 39.6% among the other serotypes [36, 37].

The porcupine dates, BD and PD have been reported to have promising in vitro antioxidant and intracellular reactive oxygen species (ROS)/reactive nitrogen species (RNS) scavenging properties, and this might be ascribed to the high content of tannins in those porcupine dates [11]. Furthermore, these tannins in porcupine dates were believed to inhibit the proliferation of the colon cancer cells in vitro by inducing apoptosis and cell cycle arrest in the cells [12]. To study whether tannins in the porcupine dates are bioactive against dengue infection, we have isolated the tannin fractions from MBD and MPD for further testing. Interestingly, both isolated tannin fractions of porcupine dates (BDTF and PDTF) exerted a dose-dependent inhibition on DENV-2 infection in Vero cells. Besides that, both BDTF and PDTF demonstrated similar and higher virucidal activity against DENV-2 when compared to their methanol crude extracts. In virucidal assay, which is determining the viral activity after direct binding of virus to porcupine dates extract, the IC_50_ of all extracts except PDTF were found to be at least 1.5-fold lower than the one observed in virus yield inhibition assay, indicating that the anti-dengue activity exhibited by those compounds was mainly due to its virucidal effect on the virus particles. Apparently, the antiviral effectiveness of these porcupine dates was higher when they interacted directly with the DENV-2 particles, thus suppressing the infectivity of DENV-2 by inactivating the extracellular DENV-2 particles.

In DENV, the envelope glycoprotein (E protein) on the viral surface is crucial for virus attachment and entry [38], and earlier study by Chen et al. [21] has proved that dengue virus infectivity is highly dependent on the binding of E protein onto heparan sulfate on host cell. Based on these findings, it is highly deemed that the extracts bind to the E protein on DENV-2 particles and thus inhibit the attachment of virus to the host cells, preventing virus infection. To ascertain this, virus attachment assay was conducted and confirmed that the porcupine dates extracts inhibited the DENV-2 infection by blocking virus attachment to the host cells. Moreover, the results of the virucidal and attachment assays correlated well with each other in term of antiviral effect, in which the porcupine dates extracts particularly the tannin fractions exerted the greatest antiviral activity against DENV-2. In both assays, the BDTF and PDTF showed similar antiviral effect as observed in reference compounds, the FT and ST tannic acids. High inhibition on the attachment of DENV-2 by heparin at low concentration (1–200 µg/mL) in Vero and BHK cells has been reported [13, 22]. However, in this study, heparin appears to be less effective in abrogating DENV-2 infection at 800 µg/mL with respect to virus attachment. This could be due to the different strain of DENV-2 being used; the degree of DENV-2 inhibition by heparin might be varied when tested on different strains of DENV-2. As the DENV-2 used in this study was isolated from a patient with severe dengue, it is highly likely that the DENV-2 strain was less susceptible to the heparin treatment.

The antiviral activity ascribed to the interaction between the extracts and host cells was also investigated; pre-treating the cells with BDTF prevented the DENV-2 infection by at least 75% and similar result was observed for FT tannic acid. This observation might be due to the presence of specific chemical structure in the FT tannic acid that is similar to the one in BDTF. It could be implied that the tannins in porcupine dates might interfere the host cell’s receptors, which are essential for DENV-2 attachment to the host cells, preventing the initiation of virus infection [21]. A difference in the effect of pre-treatment was observed in the reference compounds, ST and FT tannic acids, in which only 9.8% of virus inhibition was observed in ST in contrast to the 75.1% of virus inhibition by FT. This might be attributed to the differences in the chemical composition between ST and FT tannic acids viz. the absence of C-H functional group at wavenumbers of 2926–2929 cm^−1^ in ST tannic acid (Table 1), which is found in BDTF or PDTF.

Taken together, these results suggest that tannins in porcupine dates could be the key compounds that contribute to the antiviral effect of porcupine dates extracts against DENV-2 infection. It appears likely that the tannins in porcupine dates exerted its antiviral activity by acting on the virus particles and host cells at the early stage of DENV-2 replication, thereby suppressing the initiation of virus infection.

## Conclusions

In conclusion, porcupine dates are proven to be potentially effective in abrogating DENV-2 infection in vitro. This anti-dengue property of porcupine dates is most likely attributed to its tannin compounds. The findings in this study signify the potential of porcupine dates as an alternative natural antiviral agent against DENV. Nonetheless, further investigation on the antiviral activity of porcupine dates dengue infection in vivo and against other dengue serotypes is warranted. Besides that, the relationship and kinetics of the drugs in interacting with DENV replication is worthwhile to be further elucidated in future.

## Data Availability

All data generated or analysed during this study are included in this published article.

## Abbreviations

DENV-2: Dengue virus serotype 2
BD: Black date
PD: Powdery date
BDTF: Tannin fraction of BD
PDTF: Tannin fraction of PD
MBD: Methanol crude extract of BD
MPD: Methanol crude extract of PD
NMR: Nuclear magnetic resonance
SI: Selectivity index
FFU: Foci-forming units
MNTC: Maximum non-toxic concentration
